# Improving heart function by modulating myocardiocyte autophagy: a possible novel mechanism for cardiovascular protection of high-density lipoprotein

**DOI:** 10.1186/1476-511X-13-163

**Published:** 2014-10-22

**Authors:** Fan Wang, Ping Ye

**Affiliations:** Department of Geriatric Cardiology, Chinese PLA General Hospital, Beijing, 100853 China

**Keywords:** Autophagy, Reconstituted high-density lipoprotein, Sphingosine-1-phosphate, Heart function

## Abstract

**Background:**

High-density lipoprotein (HDL) has been shown to confer cardiovascular protection in clinical and epidemiologic studies. Emerging evidence suggests that many of the cardioprotective functions of HDL may be due to the phospholipid sphingosine-1-phosphate (S1P).

**Presentation of the hypothesis:**

HDL-S1P binds to S1P receptors in the heart, activating PI3K/Akt signaling and myocyte survival. PI3K/Akt is a classic signaling modulator of autophagy. Excessive autophagy due to cell death and cardiomyocyte loss may contribute to impaired heart function during pressure overload-induced heart failure. Therefore, we hypothesize that HDL-S1P may suppress excessive autophagy of cardiomyocytes through activation of PI3K/Akt signaling. Further, reconstituted HDL (including S1P) may protect heart function during pressure overload-induced heart failure.

**Testing the hypothesis:**

We will design the following experiments to test this hypothesis. (1) We will treat cells and mice with PI-3 kinase inhibitors to examine if HDL-S1P downregulates expression of Autophagy-related genes (ATGs) and proteins via activation of PI3K/Akt signaling. (2) We will use siRNA against S1P receptors or inhibitors of S1P receptors to determine which types of S1P receptors participate in this mechanism. (3) We will also examine if reconstituted HDL (including S1P) improves heart function during pressure overload-induced heart failure by suppressing excessive autophagy of cardiomyocytes through activation of PI3K/Akt signaling.

**Implications of the hypothesis:**

Understanding the autophagy signaling pathway modulated by HDL-S1P will make a major contribution to the field by identifying a novel mechanism for cardiovascular protection of high-density lipoprotein. Further, using reconstituted HDL to improve heart function would provide a novel therapeutic approach for pressure overload–induced heart failure.

## Background

Despite considerable improvements in medical care over the past several decades, atherosclerotic cardiovascular diseases, including coronary heart disease (CHD) and stroke, remain a major public health challenge. In fact, atherosclerotic cardiovascular diseases are responsible for nearly 50% of all deaths and are the main cause of disease burden. Post-hoc analyses of prospective trials in patients with acute coronary syndrome and stable CHD reveal that elevated plasma triglyceride levels and low plasma concentrations of high-density lipoprotein cholesterol (HDL-C) are intimately associated with high cardiovascular risk; this risk was observed even at or below the recommended low-density lipoprotein cholesterol levels [1, 2]. Furthermore, HDL-C concentrations and cardiovascular risk have been shown to have an inverse relationship in clinical and epidemiologic studies.

Cardiovascular protection of HDL has been explored. The anti-atherogenic functions of HDL are mainly mediated by reverse cholesterol transport (RCT). Moreover, there is clear evidence that HDL particles exert pleiotropic effects on anti-inflammatory, anti-oxidative, anti-apoptotic, and vasodilatory properties [3–5]. Apo A-I is a major apolipoprotein of HDL and functions as an important bioactive cardioprotective component [6]. Emerging evidence suggests that many of the cardioprotective functions of HDL, such as vasodilation, angiogenesis and endothelial barrier function, protection against ischemia/reperfusion injury, and inhibition of atherosclerosis, may be due to the phospholipid sphingosine-1-phosphate (S1P) [7, 8]. HDL-bound S1P (HDL-S1P) plays a role in HDL cardiac protection and represents a potential target for therapeutic interventions.

## Presentation of the hypothesis

### Direct effects of HDL-S1P on the heart

S1P is a bioactive lysophospholipid that regulates many important cellular processes. The major source of plasma S1P is from blood cells (mainly erythrocytes, platelets, and leukocytes) [9, 10]. Most circulating S1P is not free; instead, it is bound to plasma proteins, which seem to “buffer” S1P. The majority (50–70%) of total plasma S1P is transported by HDL, especially HDL3 particles [11], and approximately 30% of total plasma S1P is transported by albumin. Several studies suggest differences in the functions of HDL-linked S1P and albumin-linked S1P; S1P in the former form has been proposed to exert anti-atherosclerotic functions [12]. The percentage of S1P transported in plasma lipoproteins may be positively correlated with HDL-C concentrations. This suggests that individuals with high HDL-C levels may have high HDL-S1P levels, which further supports the role of S1P as a mediator of the protective effects of HDL against atherogenesis [13]. Furthermore, Theilmeier et al. suggested that HDL and S1P may attenuate the infarction size of an in-vivo mouse model of myocardial ischemia/reperfusion by inhibiting inflammatory neutrophil recruitment and cardiomyocyte apoptosis in the infarcted area [14].

In addition to the indirect cardioprotective effects of HDL, experimental myocardial infarction studies indicate that HDL also exerts direct cardioprotection mediated by S1P. The first report to attribute the direct cardioprotective effects of HDL on S1P showed that HDL protected mouse cardiomyocytes from hypoxia-reoxygenation through HDL-S1P [15]. Many S1P actions are mediated through subtypes of S1P G-protein-coupled receptors, which comprise S1P1–5. S1P binding to S1P1, 2, or 3 receptors in the heart activates downstream signaling pathways that promote myocyte survival [16]. FTY720 (Fingolimod), a S1P1,3–5-R pan-agonist, was recently approved by the FDA in 2010 as the first orally active drug for the treatment of relapsing-remitting MS. FTY-720 is able to prevent the initiation of cardiac hypertrophy. FTY-720 profoundly reverses existing hypertrophy/fibrosis through negative regulation of NFAT activity in cardiomyocytes through Gi signaling and reduction of periostin expression in the extracellular matrix, which renders a favourable milieu for myocytes, leading to improved cardiac performance. Tao R et al. reported that myocyte survival is abrogated by the PI-3 kinase inhibitor wortmannin. The PI3K/Akt signaling pathway is activated by S1P-associated HDL via the S1P3 receptor [17]. Furthermore, in neonatal rat ventricular myocytes, native and reconstituted HDL were protective against doxorubicin-induced apoptosis due to the S1P component of HDL [18]; the cardioprotection was mediated by the S1P2 receptor, ERK1/2, and STAT3.

### Autophagy and pressure-loading heart failure

In contrast to cell survival, there are three types of cell death: necrosis, apoptosis, and autophagy.

Autophagy is a housekeeping mechanism that removes aberrant and dysfunctional molecules and organelles from cells. It is a catabolic trafficking pathway for bulk destruction and turnover of long-lived proteins and organelles via regulated lysosomal degradation. Autophagy-related genes (ATGs) control the process of autophagy. Beclin-1 (Atg6) is needed for vesicle nucleation of autophagosomes. Vesicle elongation requires two conjugation pathways. One of these involves microtubule-associated protein 1 light chain 3 (LC3 or Atg8), which is converted from its soluble form into a vesicle-associated form during the process of elongation. The elongation of the isolation membrane is followed by maturation of the autophagosome, which fuses with a lysosome, thereby generating an autophagolysosome or autolysosome. Class I PI3K/Akt is the classic signaling pathway to regulate expression of ATGs; activation of PI3K/Akt increases expression of ATGs.

In the heart, autophagy occurs constitutively at low basal levels to perform housekeeping functions that maintain cardiac function and morphology [19]. However, autophagic processes in cardiomyocytes are up-regulated in response to altered internal needs and environmental stressors, such as removal of protein aggregates, ATP depletion (e.g., during starvation), and oxidative damage (e.g., via reactive oxygen species) [20, 21]. These observations strongly suggest that autophagy is a survival mechanism. Indeed, it has been demonstrated that autophagy plays a critical role in the maintenance of ventricular function during starvation in the adult [22]. On the other hand, the occurrence of autophagic structures in dying cells of different organisms has led to the hypothesis that autophagy may also play a causative role in stress-induced cell death [23]. It has become clear that failing hearts simultaneously exhibit apoptosis, necrosis, and autophagy [24]. In particular, autophagy has been observed in the failing human heart; upregulation of this process has also been reported in animal models of pressure overload-induced heart failure [25]. The extent of autophagic flux can increase to maladaptive levels during load-induced heart failure. Excessive autophagy leads to autophagic cell death and loss of cardiomyocytes, which may contribute to heart failure [26]. Autophagy functions predominantly as a pro-survival mechanism during nutrient deprivation or other forms of cellular stress. However, when autophagy is strongly induced, the autophagic machinery may also be used for self-destruction. Hence, when autophagic cell death occurs in cardiac cells, it may contribute to the progress of heart failure.

### Hypotheses

HDL-S1P binds to S1P receptors in the heart, activating PI3K/Akt signaling and subsequent myocyte survival. PI3K/Akt is a classic signaling modulator of autophagy. Excessive autophagy due to cell death and loss of cardiomyocytes may impair heart function during pressure overload-induced heart failure. Our preliminary results indicated that reconstituted HDL (including S1P) suppressed autophagy of rat cardiomyocytes in the presence of phenylephrine at serum-deprivation (unpublished observations). Therefore, we hypothesize that HDL-S1P suppresses excessive cardiomyocyte autophagy by activating PI3K/Akt signaling. Further, we propose that reconstituted HDL (including S1P) protects heart function during pressure overload-induced heart failure.

### Testing the hypothesis

We will design the following experiments to test this hypothesis. (1) We will treat neonatal rat cardiomyocytes and mice with PI-3 kinase inhibitors to examine if HDL-S1P downregulates expression of Autophagy-related genes (ATGs) and proteins via activation of PI3K/Akt signaling. (2) We will use siRNA against S1P receptors or inhibitors of S1P receptors to determine which types of S1P receptors participate in this mechanism. (3) We will also examine if reconstituted HDL (including S1P) improves heart function during pressure overload–induced heart failure by suppressing excessive autophagy of cardiomyocytes through activation of PI3K/Akt signaling.

### Implications of the hypothesis

Understanding the autophagy signaling pathway modulated by HDL-S1P will make a major contribution to the field by identifying a novel mechanism for cardiovascular protection of high-density lipoprotein. Further, using reconstituted HDL (including S1P) to improve heart function would provide a novel therapeutic approach for pressure overload–induced heart failure.

## Abbreviations

HDL: High-density lipoprotein
S1: sphingosine-1-phosphate
HDL-S1P: HDL-bound S1P
ATGs: Autophagy-related genes.
